# *Porphyromonas gingivalis* Outer Membrane Vesicles‐Associated DNA Triggers Inflammation by Inducing IL‐6 in Astrocytes

**DOI:** 10.1111/omi.70030

**Published:** 2026-04-01

**Authors:** Ayu Takai, Kaya Yoshida, Yuka Hiroshima, Ayu Ikuta, Mariko Seyama, Yuta Uemura, Hiromichi Yumoto, Kazumi Ozaki

**Affiliations:** ^1^ Department of Oral Healthcare Promotion Graduate School of Biomedical Sciences Tokushima University Tokushima Japan; ^2^ Department of Oral Microbiology Graduate School of Biomedical Sciences Tokushima University Tokushima Japan; ^3^ Department of Periodontology and Endodontology Graduate School of Biomedical Sciences Tokushima University Tokushima Japan

**Keywords:** Alzheimer's disease, astrocytes, interleukin‐6, outer membrane vesicles, periodontal diseases, *Porphyromonas gingivalis*

## Abstract

*Porphyromonas gingivalis* (Pg), a key pathogen in periodontal disease, is suggested to be involved in the progression of Alzheimer's disease (AD); however, the molecular mechanism remains unclear. We previously reported that Pg‐released outer membrane vesicles (OMVs) were detected in the brains of mice after intraperitoneal administration. We here investigated the effects of Pg OMVs on astrocytes, the most abundant glial cells in the central nervous system, which are involved in neuroinflammation. We demonstrated that Pg OMVs increased the expression of interleukin‐6 (IL‐6) mRNA, which is associated with the pathogenesis of AD, in a Toll‐like receptor (TLR)2/4‐independent manner in human astrocyte SVG p12 cells. Whole‐genome sequencing revealed that Pg OMVs contained Pg genomic DNA, which was critical for IL‐6 mRNA induction. The tracking of fluorescent‐labeled Pg OMV‐associated DNA revealed that Pg OMVs were transported into SVG p12 cells. Endocytosis inhibitors attenuated IL‐6 mRNA expression induced by Pg OMVs, suggesting that the incorporation of Pg OMVs by endocytosis is important for IL‐6 mRNA induction. Furthermore, the incorporated Pg OMV‐associated DNA increased IL‐6 mRNA expression via the TLR9 pathway. Our study advances understanding of the role of Pg OMVs, which may contribute to the onset and progression of AD in periodontal disease.

## Graphical abstract

Pg releases OMVs which contain Pg genomic DNA. The Pg OMVs‐associated DNA is incorporated into astrocytes via endocytosis and increases IL‐6 mRNA expression via TLR9. These effects of Pg OMVs may induce neuroinflammation, which could contribute to the onset and progression of AD in periodontal disease.

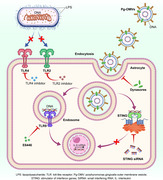

## 1 Introduction

Periodontal diseases have been identified as significant risk factors for developing Alzheimer's disease (AD). Many clinical reports have shown that elderly individuals with chronic periodontal disease exhibit increased accumulation of amyloid β (Aβ) in the brain (Kamer et al. 2015), as well as the onset of memory impairment (Noble et al. 2009). However, the molecular mechanisms underlying these phenomena remained unclear for a long time. *Porphyromonas gingivalis* (Pg), a Gram‐negative bacterium and a key periodontal pathogen (Darveau et al. 2012), has recently been the focus of research into AD, because Pg DNA and its specific protease, gingipains, have been detected in the brains of patients with AD (Dominy et al. 2019). Rats orally infected with Pg also showed AD pathogenesis, such as Aβ accumulation and tau phosphorylation, leading to cognitive defects (Díaz‐Zúñiga et al. 2020). In fact, it is expected that the synthesized, low‐molecular‐weight gingipain inhibitor compounds will reduce neuroinflammation in patients with AD (Arastu‐Kapur et al. 2020; Kanagasingam et al. 2020 ).　These findings suggest that Pg is a risk factor for AD. However, how Pg and its components reach the brain remains unclear.

Pg releases vesicles called outer membrane vesicles (OMVs), which are approximately 150 nm in diameter and consist of an outer membrane containing most of the Pg virulence factors (Veith et al. 2014). We previously reported that Pg OMVs injected into the abdominal cavity translocated to the liver and induced glucose tolerance (Seyama et al. 2020). Pg OMVs also increased vascular permeability in vascular endothelial cells (Mekata et al. 2024) and have been detected in the brains of mice after intraperitoneal administration (Yoshida et al. 2022). Based on these findings, we hypothesized that Pg OMVs carry Pg‐derived factors to the brain and contribute to the progression of AD.

Glial cells are a general term for nervous system cells other than neurons, referring to microglia, astrocytes, and oligodendrocytes. Glial cells not only maintain and support the function of neurons but also play a crucial role in all major aspects of brain development, function, and disease (Barres 2008). Astrocytes, the most abundant glial cells in the central nervous system, induce neuroinflammation and contribute to the progression of AD (Carter et al. 2019). Astrocytes normally protect neurons by removing excess ions, neurotransmitters, and Aβ, and by forming the blood–brain barrier (BBB) together with vascular endothelial cells and pericytes (Luissint et al. 2012). On the other hand, in AD brain tissue, reactive astrocytes were found around Aβ and tau (Lemoine et al. 2017; Serrano‐Pozo et al. 2011). Reactive astrocytes release a variety of pro‐inflammatory mediators, and chronic neuroinflammation may induce synaptic dysfunction and BBB dysregulation, which promote cognitive defect in AD (Bettcher and Kramer 2014; Paidlewar et al. 2024).

Among the pro‐inflammatory mediators, interleukin‐6 (IL‐6) plays an important role in neuroinflammation. IL‐6 levels are elevated in the cerebrospinal fluid (CSF) and plasma of patients with mild cognitive impairment (MCI) and AD (Brosseron et al. 2014; Swardfager et al. 2010). IL‐6 is upregulated in the brain and plasma of patients with AD and inversely correlated with cognitive performance (Lyra et al. 2021). In animal models of AD, IL‐6–positive astrocytes and plaques were detected in the hypothalamus and cortex, and the neutralization of IL‐6 in the brain rescued memory deficits (Lyra et al. 2021). Although these findings suggest that IL‐6 produced by astrocytes is associated with AD pathogenesis, the relationship between periodontal diseases, Pg, and IL‐6 production in astrocytes remains unknown.

In this study, we examined the effect of Pg OMVs on IL‐6 mRNA expression in human astrocytes SVG p12 cells to investigate how periodontal disease induces neuroinflammation leading to AD progression.

## 2 Materials and Methods

### 2.1 Chemicals and Antibodies

All reagents used in this study were commercially available as follows; Toll‐like receptor (TLR)2‐IN‐C29 (Selleck Chemicals, Tokyo, Japan), TLR4‐IN‐C34 (Selleck Chemicals), Dynasore (Selleck Chemicals), E6446 (Selleck Chemicals), LPS‐RS Ultrapure (InvivoGen, San Diego, California, USA), Gene Ladder 100 (Nippon Gene, Tokyo, Japan), Gene Ladder Wide 1 (Nippon Gene, Tokyo, Japan), DNase I (Thermo Fisher Scientific, Waltham, Massachusetts, USA), S1 Nuclease (Takara Bio Inc., Otsu, Japan), QuantiFluor One dsDNA system (Promega, Madison, Wisconsin, USA), Lipofectamine RNAiMAX Reagent (Thermo Fisher Scientific), negative control siRNA and STING siRNA (Sigma–Aldrich, St. Louis, Missouri, USA). The following primary antibodies were used: anti‐STING (D2P2F) (1:1000; #13647; Cell Signaling Technology, Beverly, Massachusetts, USA) and anti‐GAPDH (14C10) (1:1000; #2118; Cell Signaling Technology).

### 2.2 Bacterial Culture

Pg American Type Culture Collection (ATCC) 33277 was cultured in Brain Heart Infusion medium (BD Biosciences, Franklin Lakes, New Jersey, USA) containing 0.5% yeast extract (BD Biosciences), 10 µg/mL hemin (Wako Chemicals, Osaka, Japan), and 1 µg/mL 2‐methyl‐1,4‐naphthoquinone (Tokyo Chemical Industry, Tokyo, Japan). The Pg was cultured in an anaerobic jar at 37°C until the absorbance at 600 nm reached between 0.8 and 1.0, after which it was used for the experiment.

### 2.3 Pg OMVs Isolation

Pg ATCC 33277 culture medium was centrifuged at 2800 × *g* for 15 min at 4°C and the supernatant was filtered with a 0.2‐µm syringe filter, and then concentrated using an Ultra‐15 Centrifugal Filter Device (Merck Millipore, Burlington, Massachusetts, USA). OMVs were isolated from the concentrate using Total Exosome Isolation Reagent (Thermo Fisher Scientific, Waltham, Massachusetts, USA) according to the manufacturer's protocol. The protein concentration of OMVs used for the experiments was measured using the Bradford Protein Assay Reagent (Bio‐Rad Laboratories Inc., Hercules, California, USA) and adjusted immediately before use. The obtained Pg OMVs were stored at 4°C and used for subsequent experiments.

### 2.4 Cell Culture

Human brain astroglial cells (SVG p12) were obtained from the ATCC (CRL‐8621) and cultured in minimum essential medium Eagle (EMEM) (Sigma–Aldrich, M4655) with 10% fetal bovine serum. The cells were cultured at 37°C under a humidified atmosphere of 5% CO_2_ and used for experiments after reaching 70%−80% confluence.

### 2.5 Inhibitor Treatment

After incubating the SVG p12 cells with various inhibitors for 30 min, the Pg OMVs were added at a concentration of 5000 ng/mL and incubated for 120 min. The concentrations of each inhibitor were as follows: 10 µM for TLR2‐IN‐C29 and TLR4‐IN‐C34, 80 µM for Dynasore, and 10 µM for E3446.

### 2.6 RNA Isolation and Real‐Time PCR

Total RNA was isolated from the cells using ISOGEN (Nippon Gene). cDNA was synthesized using the ReverTra Ace qPCR RT Master Mix (TOYOBO, Kyoto, Japan), and real‐time PCR was performed using a 7300 Real‐Time PCR system (Applied Biosystems, Carlsbad, California, USA) and THUNDERBIRD SYBR qPCR Mix (TOYOBO). The following primer sequences were used: human IL‐6 (NM_000600.4), Forward: 5'‐AAGCCAGAGCTGTGCAGATGAGTA‐3', Reverse: 5'‐TGTCCTGCAGCCACTGGTTC‐3'; human GAPDH (NM_002046.7), Forward: 5'‐GCACCGTCAAGGCTGAGAAC‐3', Reverse: 5'‐TGGTGAAGACGCCAGTGGA‐3'; human STING (NM_198282.4), Forward: 5'‐GGATATCTGCGGCTGATCCTG‐3', Reverse: 5'‐AGCAGGTTGTTGTAATGCTGATTG‐3'.

### 2.7 DNA Isolation and Measurement

DNA was isolated from Pg OMVs using a Quick‐DNA Miniprep Plus Kit (Zymo Research, USA) according to the manufacturer's protocol. Subsequently, the obtained DNA was quantified using QuantiFluor One dsDNA system (Promega, E4871), and then DNA concentration was measured by Quantus Fluorometer (Promega). The prepared DNA was analyzed using 0.8% agarose gels in 1× TAE (Tris‐acetate‐ethylenediaminetetraacetic acid [EDTA]) buffer containing ethidium bromide, which were then photographed using transillumination with UV light.

### 2.8 Whole Genome Sequencing

Whole‐genome sequencing of the DNA extracted from Pg and Pg OMVs were provided by Rhelixa Inc. (Tokyo, Japan, ID: RH23056888). After checking DNA quality, libraries were prepared for sequencing using the NEBNext Ultra DNA Library Prep Kit (New England Biolabs, Ipswich, Massachusetts, USA). Paired‐end short reads (150 bp) were generated using an Illumina NovaSeq 6000 (Illumina, San Diego, California, USA). *Porphyromonas gingivalis* ATCC 33277 (https://www.ncbi.nlm.nih.gov/genome/714?genome_assembly_id = 300313) genome sequence was used as a reference genome. PyCirclize software (v0.5.1) was used to generate a figure showing read counts per 1000 bp region (Rhelixa Inc., RH23056888).

### 2.9 DNase Treatment

Two units of DNase I were added to the Pg OMVs and incubated at 37°C for 30 min. Then 0.5 M EDTA was added and incubated at 65°C for 10 min to inactivate DNase activity.

### 2.10 Pg OMVs Labeling

DNA from Pg OMVs was labeled with SYTO 61 Red Fluorescent Nucleic Acid Stain (Thermo Fisher Scientific, 10217202) according to the manufacturer's protocol. Briefly, 50 µg of Pg OMVs were incubated with 5 µM SYTO 61 in phosphate‐buffered saline (PBS) for 30 min under protected light and then washed with PBS by centrifugation at 145,000 × *g* for 80 min. After two washes, the obtained pellet was resuspended in PBS. Proteins in the samples were quantified and used in the experiments at a concentration of 5000 ng/mL. To verify labeling efficiency, the fluorescence of SYTO 61–labeled Pg OMVs was measured at 600 nm (excitation) and 645 nm (emission) using an Infinite M200 PRO quad4 monochromator (Tecan, Männedorf, Switzerland) and observed under a Nikon ECLIPSE Ti‐U microscope (Nikon, Tokyo, Japan).

### 2.11 Immunofluorescence Analysis

SVG p12 cells were treated with SYTO 61–labeled Pg OMVs at a concentration of 5000 ng/mL for 120 min, and then the unincorporated SYTO 61–labeled Pg OMVs were washed away with PBS. Subsequently, the nucleus was stained with 2 µg/mL Hoechst 33342 for 20 min in Hanks’ Balanced Salt Solution (HBSS). After washing the cells, 2.5 µg/mL CellMask Plasma Membrane Stain Green (Thermo Fisher Scientific, C37608) was applied for 5 min to stain the plasma membrane, followed by treatment with 100 nM Mito Tracker Green FM (Thermo Fisher Scientific, 490516) for 30 min to visualize mitochondria. After washing off the excess dye, the cells were observed under a Nikon A1R confocal microscope (Nikon) while immersed in HBSS. The intensity profiles at each wavelength (DAPI, 405 nm; FITC, 488 nm; TRITC, 561 nm) were analyzed using an A1R confocal microscope (Nikon).

### 2.12 siRNA

SVG p12 cells were seeded at 100,000 cells/mL and transfected with siRNA the following day at 80% confluence. Cells were transfected with 50 nM STING siRNA or negative control siRNA (Sigma–Aldrich, Oligo# 3024347668‐000020, 3024347668‐000030) using Lipofectamine RNAiMAX Reagent, following the manufacturer's protocol.

### 2.13 Western Blotting

Cells were collected and scraped in lysis buffer (1 mM dithiothreitol (DTT), 0.2 mM phenylmethylsulphonyl fluoride (PMSF), 1 µg/mL leupeptin, 4 µg/mL aprotinin, and 50 mM NaF), sonicated for 20 s, and then centrifuged at 12,000 × *g* for 30 min. The supernatants were prepared with sample buffer (1 M Tris‐HCl pH 6.8, 0.5 M DTT, 10% SDS, 50% glycerol, and 0.02% bromophenol blue) and heated for 10 min at 100°C. Samples were separated using sodium dodecyl sulfate‐polyacrylamide gel electrophoresis (SDS‐PAGE) and transferred to polyvinylidene difluoride membranes (Immobilon‐P; Millipore, Temecula, California, USA).

The membranes were blocked with 1% skim milk in TBS‐T for 1 h, and incubated with primary antibodies for 12 h at 4°C. The membranes were washed with TBS‐T for 30 min and subsequently incubated with anti‐rabbit IgG antibody conjugated with horseradish peroxidase (HRP) (1:10,000, #7074, Cell Signaling Technology) for 45 min. Signals were detected using Western Blot Chemiluminescence HRP Substrate (Takara Bio Inc., Otsu, Japan).

### 2.14 Statistical Analysis

Statistical analyses were performed using Statcel2 software (OMS Publishing, Tokorozawa, Japan). The normal distribution of the data was first examined using the chi‐square test. Variables with a normal distribution were analyzed using Student's *t*‐test. All data are expressed as mean ± standard deviation (SD). *p* < 0.05 was considered statistically significant. All experiments were repeated at least three times to ensure similar results.

## 3 Results

### 3.1 Pg OMVs Increase IL‐6 mRNA in TLR2/4‐Independent Manner in SVG p12 Cells

**FIGURE 1:**
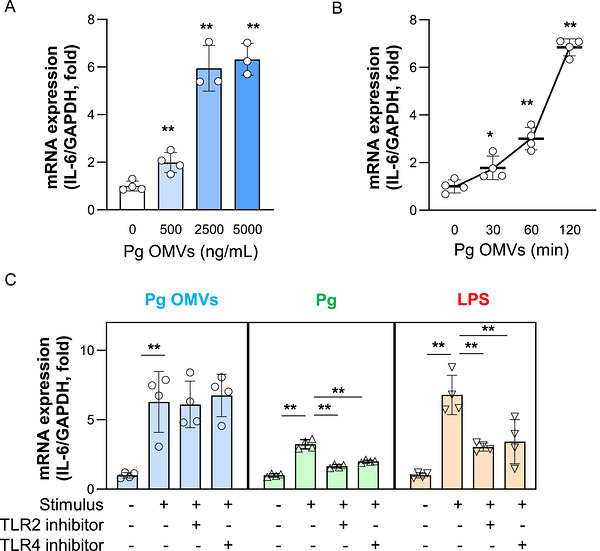
Pg OMVs increase IL‐6 mRNA in TLR2/4‐independent manner in SVG p12 cells. (A, B) The change in IL‐6 mRNA expression by Pg OMVs in SVG p12 cells. SVG p12 cells were treated with (A) various concentration of Pg OMVs for 120 min or (B) with 5000 ng/mL Pg OMVs for indicated periods. (C) Effect of TLR2 and TLR4 inhibition on Pg OMVs‐induced IL‐6 mRNA level. SVG p12 cells were pretreated with TLR2‐IN‐C29 (10 µM) and TLR4‐IN‐C34 (10 µM) before treatment with Pg OMVs (5000 ng/mL), Pg (100 MOI), and *E. coli* LPS (1 µg/mL) for 120 min. Student's *t*‐test was used for statistical analysis. **p < 0.01 compared with no‐treated cells. *n* = 4. All data represent the mean ± SD from results of 3–4 independent experiments.

Human astrocyte SVG p12 cells were cultured and treated with various concentrations of OMVs extracted from *P. gingivalis* ATCC 33277 strain (0–5000 ng/mL) for 120 min, and IL‐6 mRNA levels were assessed using real‐time PCR. Treatment with Pg OMVs increased IL‐6 mRNA levels in SVG p12 cells (Figure 1A). Next, SVG p12 cells were treated with 5000 ng/mL Pg OMVs for 120 min. The IL‐6 mRNA levels increased 30 min after treatment and reached a maximum at 120 min (Figure 1B). Thus, the conditions for Pg OMV treatment of SVG p12 cells were set at a concentration of 5000 ng/mL and time of 120 min for subsequent experiments.

Since we confirmed that Pg OMVs are rich in lipopolysaccharides (LPS) (Seyama et al. 2020) and LPS and embedded lipoproteins are TLR2 and TLR4 agonists, Pg OMVs may induce IL‐6 mRNA by binding to TLR2 and/or 4 on the SVG p12 plasma membrane. To verify this possibility, we pretreated SVG p12 cells with TLR2‐IN‐C29 (a TLR2 inhibitor) and TLR4‐IN‐C34 (a TLR4 inhibitor) for 30 min before Pg OMVs treatment, and then assessed IL‐6 mRNA levels. As positive controls that bind to TLR2/4 and transduce signaling, Pg and LPS from *E. coli* were also treated. Both Pg and LPS increased IL‐6 mRNA expression and this induction was significantly attenuated by TLR2/4 inhibition. In contrast, the IL‐6 levels increased by Pg OMVs were not affected by TLR2 and 4 inhibitors, suggesting that Pg OMVs induced IL‐6 mRNA in a TLR2/4‐independent manner (Figure 1C).

In this study, the concentration of Pg OMVs was quantified based on protein concentration. However, this method of quantification may detect contamination introduced during the extraction process. To confirm that we were indeed extracting Pg OMVs, we measured and compared protein concentration, particle size, and particle concentration. Additionally, to confirm the reproducibility of the extraction method, measurements were performed on three Pg OMVs extracted using the same method on different days. The results showed that sufficient quantities of Pg OMVs were extracted and that the extraction technique did not vary (Figure S1).

### 3.2 DNA in Pg OMVs Induce IL‐6 mRNA in SVG p12 Cells

**FIGURE 2:**
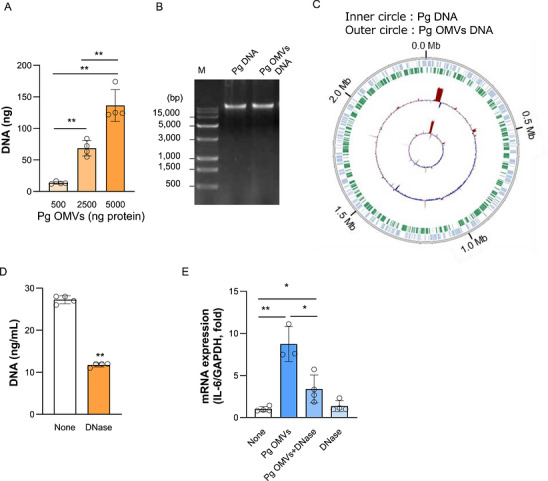
DNA in Pg OMVs induce IL‐6 mRNA in SVG p12 cells. (A) DNA content in Pg OMVs. DNA was isolated from each protein content of Pg OMVs. (B) DNA (50 ng) isolated from Pg (Pg DNA) and Pg OMVs (Pg OMVs DNA) was subjected to electrophoresis and stained with ethidium bromide. M, marker. (C) Whole genome sequences of Pg OMVs DNA and Pg DNA. Inner circle, read counts for Pg DNA; outer circle, Pg OMVs DNA. Blue‐gray plots, the sequence regions on the positive strand of the reference genome. Green plots, the sequence regions on the negative strand. Red plots, the above‐average read counts. Blue plots, below‐average read counts. (D) Decrease of Pg OMVs containing DNA by DNase. (E) Effects of DNA on Pg OMVs‐induced IL‐6 mRNA levels. SVG p12 cells were treated with Pg OMVs or with DNase‐treated Pg OMVs (Pg OMVs + DNase). As a negative control, the group treated with DNase only in a buffer without Pg OMVs (DNase) is shown. Student's *t*‐test was used for statistical analysis. *p < 0.05; **p < 0.01 compared with indicated group. *n* = 4. All data represent the mean ± SD from results of three independent experiments.

Bacterial DNA contains unmethylated CpG motifs which bind to TLR9 and induce innate immune system (Hemmi et al. 2000), and DNA prepared from Pg has been reported to stimulate macrophages and gingival fibroblasts to produce IL‐6 (Nonnenmacher et al. 2003). We, therefore, investigated the involvement of DNA contained in Pg OMVs. DNA levels (ng/mL) in Pg OMVs at protein of 500, 2500, and 5000 ng were measured and converted to DNA content. DNA content increased in a Pg OMVs concentration‐dependent manner (Figure 2A). DNA was extracted from Pg OMVs and from Pg whole bacteria as a positive control. After electrophoresis, the DNA was stained with ethidium bromide as shown in Figure 2B; Pg OMVs contained DNA that is similar to Pg whole bacteria (Figure 2B).

To investigate which DNA is packaged from Pg to Pg OMV, we sequenced the whole genome of Pg OMVs and Pg whole bacteria to compare their DNA. The total number of reads obtained was 10,824,234 for Pg DNA and 10,001,586 for Pg OMVs DNA. Of these, 10,672,459 and 9,833,807 reads were mapped to the reference genome (genome sequence of Pg ATCC 33277), respectively, with mapping rates of 99.98% in Pg DNA and 99.96% in Pg OMVs DNA. The circular plot in Figure 2C shows the read counts for Pg DNA in the inner circle and Pg OMVs DNA in the outer circle. There was no difference in the read counts mapped to each position on the reference genome between Pg OMVs DNA and Pg DNA, suggesting that Pg OMVs DNA was nearly identical to Pg DNA (Figure 2C). This also suggests that Pg genomic DNA entered is present in Pg OMVs.

Next, we examined whether the DNA contained in Pg OMVs was involved in IL‐6 mRNA induction. To remove DNA from the surface of Pg OMVs, the Pg OMVs were treated with DNase, which is impermeable to membranes. Analysis using a transmission electron microscope has demonstrated that DNase cannot permeate the membrane of OMVs and therefore only removes DNA from their surface (Bitto et al. 2017). Treatment with DNase significantly reduced the concentration of DNA in the Pg OMVs (Figure 2D), suggesting that DNA present on the surface of OMVs was removed by the membrane‐impermeable DNase. Treatment of SVG p12 cells with DNase‐treated Pg OMVs (from which surface DNA had been removed) resulted in significantly increased IL‐6 mRNA expression compared to the group with nothing added and is shown as “None”. However, compared to DNase‐untreated Pg OMVs (containing surface DNA), the extent to which DNase‐treated Pg OMVs increased IL‐6 mRNA expression was significantly lower (Figure 2E). These results suggest that at least the DNA on the surface of Pg OMVs is involved in inducing IL‐6 mRNA in SVG p12 cells.

### 3.3 Pg OMVs DNA Is Delivered Into SVG p12 Cells

**FIGURE 3:**
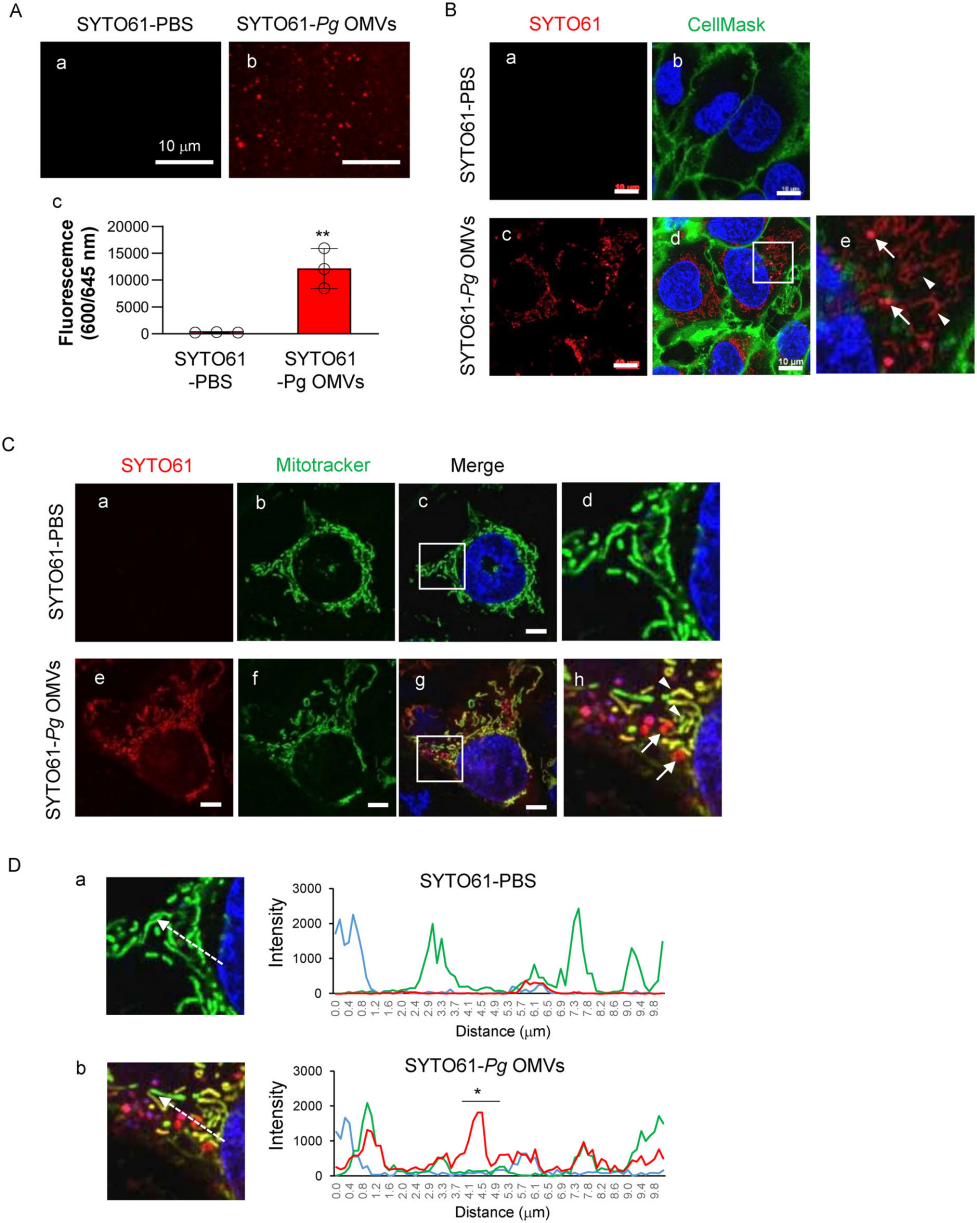
Pg OMVs DNA is delivered into SVG p12 cells. (A) Pg OMVs (10 µg) were labeled with SYTO 61 (SYTO 61–Pg OMVs DNA). As negative control, total volume of PBS was also labeled with SYTO 61 (SYTO 61–PBS). (a, b) Each sample were observed under fluorescent microscopy, and (c) measured fluorescence. Scale bar, 10 µm. Student's *t*‐test was used for statistical analysis. **p < 0.01. *n* = 3. All data represent the mean ± SD from results of three independent experiments. (B) SVG p12 cells were treated with (a, b) SYTO 61–PBS or (c, d, e) SYTO 61–Pg OMVs DNA for 120 min. (b, d, e) The plasma membrane was labeled with CellMask Green (green), and the nuclei were stained with Hoechst 33342 (blue). (e) High‐magnification merged images of square in (d) are shown. The arrows indicate granular structures, and the arrowhead represents the structures that appeared to be mitochondria. Scale bars indicate 10 µm. (C) In cells treated with the same as (B), (a, e) SYTO 61–Pg OMVs DNA (red) and (b, f) mitochondria (green) were visualized. (c, g) Nuclei were stained with Hoechst 33342 and the microscopic images of the same field were merged. (d, h) High‐magnification images of the merge in squares are shown. The arrows indicate SYTO 61–Pg OMVs DNA (red), and the arrowhead represents mitochondria (green and yellow). Scales indicate 10 µm. (D) The intensity profile of each fluorescence in the area is shown as the dashed arrow in (C) (d, e). Blue: Hoechst, green: FITC, red: TRITC.

We further investigated how Pg OMVs DNA affected SVG p12 cells and increased IL‐6 mRNA levels. SYTO dyes diffuse passively through the membranes of most cells and can stain nucleic acids in eukaryotic cells and Gram‐positive and Gram‐negative bacteria. We therefore used SYTO 61 to label the DNA in Pg OMVs with red fluorescence (SYTO 61–Pg OMVs). Samples in which PBS was mixed with SYTO 61 instead of Pg OMVs (SYTO 61–PBS) were used as negative controls. SYTO 61–Pg OMVs were detectable in red under a fluorescence microscope, whereas SYTO 61–PBS was not (Figure 3A,a,b). The fluorescence intensity of 10 µg of either SYTO 61–PBS or SYTO 61–Pg OMVs was measured using a plate reader, and a significantly higher fluorescence intensity was obtained with SYTO 61–Pg OMVs than with SYTO 61–PBS (Figure 3A,c). Based on these results, we concluded that the DNA in Pg OMVs was effectively labeled with SYTO 61.

Next, SVG p12 cells were treated with 5000 ng/mL SYTO 61–Pg OMVs for 120 min. Subsequently, the plasma membrane was stained with CellMask Green to clearly outline the cells. The nuclei were stained with Hoechst 33342. In cells treated with SYTO 61–Pg OMVs, red fluorescence was observed in the cytoplasmic region (Figure 3B,c,d). When the image was enlarged, the granular structures that appeared to be SYTO 61–Pg OMVs as shown in Figure 3A was observed (Figure 3B,e; arrows). Morphological structures consistent with mitochondria were detected as red fluorescence (Figure 3B,e; arrowhead). In contrast, no red fluorescence indicative of granular or mitochondria‐like structures was observed in SVG p12 cells treated with SYTO 61–PBS (Figure 3B,a,b).

Since SYTO 61 binds to the mitochondria in animal cells, we hypothesized that residual dye from the Pg OMVs label may be bound to the mitochondria. To examine this possibility, the mitochondria were visualized and compared with the red image visualized using SYTO 61. In cells treated with both SYTO 61–PBS and SYTO 61–Pg OMVs, the mitochondria were efficiently visualized around the nucleus using Mito Tracker Green (Figure 3C,b,f). Red fluorescence was observed in SYTO 61–Pg OMVs‐treated cells, but not in SYTO 61–PBS‐treated cells, as shown in Figure 3B (Figure 3C,a,e). When the red and green images were merged, the red granular structures did not overlap with the green structures, suggesting that these particle structures are not mitochondria (Figure 3C,g,h; arrows). Figure 3D shows the fluorescence intensity profiles for each dashed line in the areas shown in Figure 3C,d,h. In SYTO 61–Pg OMVs treated cells, the intensity of red fluorescence corresponding to the granular structure was different from that of mitochondrial green fluorescence (Figure 3D,b; indicated by an asterisk). These results suggest that the red granular structures represent intracellular Pg OMVs DNA labeled with SYTO 61, and that Pg OMVs DNA was delivered into the SVG p12 cells.

### 3.4 Endocytosis of Pg OMVs DNA Is Required for IL‐6 mRNA Induction in SVG p12 Cells

**FIGURE 4:**
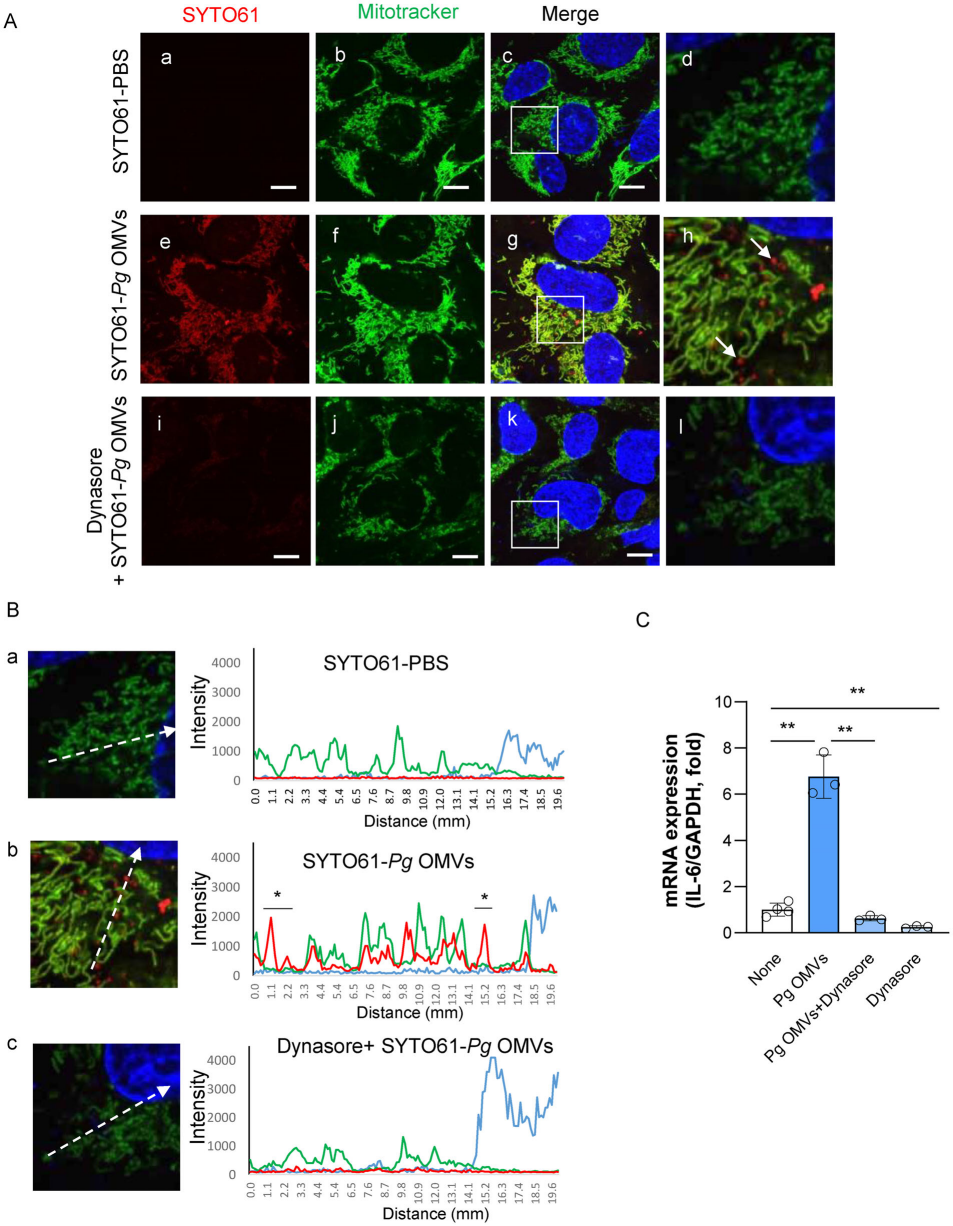
Endocytosis of Pg OMVs DNA is required for IL‐6 mRNA induction in SVG p12 cells. (A) SVG p12 cells were pretreated (i–l) with or (a–h) without 80 µM of Dynasore, and then treated with (a–d) 5000 ng/mL of SYTO 61–PBS or (e–l) SYTO 61–Pg OMVs. (a, e, i) SYTO 61–Pg OMVs (red) and (b, f, j) mitochondria (green) were visualized. Nuclei were stained with Hoechst 33342. (c, g, k) The microscopic images of the same field were merged, and (d, h, l) the area enclosed by the square is enlarged. The arrows indicate SYTO 61–Pg OMVs (red). Scales indicate 10 µm. (B) The intensity profile of each fluorescence in the area is shown as the dashed arrow in (A) (d, i, k). Blue: Hoechst, green: FITC, red: TRITC. Asterisks indicate DNA in Pg OMVs labeled with SYTO 61 (red). (C) SVG p12 cells were pretreated with 80 µM of Dynasore before treatement with 5000 ng/mL of Pg OMVs, and then IL‐6 mRNA expression was measured by real‐time PCR. **p < 0.01. *n* = 4. All data represent the mean ± SD from results of three independent experiments.

Next, we examined how Pg OMV DNA enters SVG p12 cells. Dynasore inhibits endocytosis by suppressing the GTPase activity of dynamin proteins, which is essential for the coated vesicles to detach from the membrane during clathrin‐mediated endocytosis (Macia et al. 2006). SVG p12 cells were pretreated with 80 µM Dynasore for 30 min before the treatment with SYTO 61–PBS or SYTO 61–Pg OMVs. Consistent with the results shown in Figure 3C, the DNA in SYTO 61–Pg OMVs was detected as red fluorescence in SVG p12 cells after 120 min of treatment (Figure 4A,e), whereas no red fluorescence was detected in SYTO 61–PBS‐treated cells (Figure 4A,a). When the red fluorescence image was merged with the green fluorescent mitochondrial image, DNA in Pg OMVs could be distinguished and was found in the nuclear periphery (Figure 4A,g,h; arrows). In cells pretreated with Dynasore to inhibit endocytosis, mitochondria (Figure 4A,j) and Pg OMVs DNA (Figure 4A,i) were barely detectable.

We further verified these results using the intensity profile of fluorescence along the dashed arrows in Figure 4A,d,h,l. In the SYTO 61–Pg OMVs DNA‐treated cells, red fluorescence, which did not merge with green fluorescence, was detected (Figure 4A,h). This red fluorescence was no longer detected in the cells in which endocytosis was inhibited by Dynasore (Figure 4A,l). These results were confirmed by the intensity profiles of each fluorescence signal. In SYTO 61–Pg OMVs‐treated cells, Pg OMVs DNA was distinguished from mitochondrial green fluorescence (Figure 4B,b; shown as an asterisk), whereas intracellular Pg OMVs DNA was decreased by endocytosis inhibition with Dynasore (Figure 4B,c).

We further examined whether endocytosis of Pg OMVs is necessary for increase of IL‐6 mRNA expression. The increase in IL‐6 mRNA expression by Pg OMVs was abolished by inhibiting endocytosis using Dynasore (Figure 4C), suggesting that Pg OMVs DNA, which is incorporated into SVG p12 cells via endocytosis, is involved in IL‐6 mRNA induction.

### 3.5 TLR9 Pathway Regulates IL‐6 mRNA Induced by Pg OMVs in SVG p12 Cells

**FIGURE 5:**
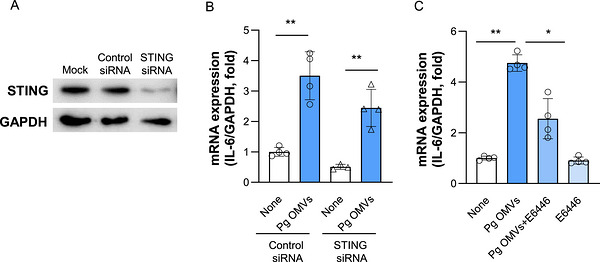
TLR9 pathway regulates IL‐6 mRNA induced by Pg OMVs in SVG p12 cells. (A) STING siRNA or control RNAi were transfected to SVG p12 cells. At 24 h after transfection, the expression ofSTING protein were analyzed by qPCR and western blot. (B) SVG p12 cells with STING knock down were treated with Pg OMVs, and then IL‐6 mRNA expression was measured by real‐time PCR. (C) Effect of TLR9 on Pg OMVs‐induced IL‐6 mRNA level. SVG p12 cells were pretreated with E6446, the inhibitor of TLR9, and then treated with Pg OMVs. The expression of IL‐6 mRNA was measured by real‐time PCR. *p < 0.05; **p < 0.01. *n* = 4. All data represent the mean ± SD from results of three independent experiments.

Bacterial DNA incorporated into cells is recognized by the stimulator of interferon genes (STING) (Zhang et al. 2020) and TLR9 pathways, which lead to the production of immune mediators. To examine the involvement of STING pathways in Pg OMVs‐induced IL‐6, we knocked down its expression using siRNAs. The results of Western blotting showed that STING expression was efficiently knocked down after siRNA treatment compared with that in the control oligo (Figure 5A). In both the STING knockdown and control oligo‐treated groups, Pg OMVs increased IL‐6 mRNA expression, suggesting that STING knockdown does not play a significant role in IL‐6 mRNA induction by Pg OMVs (Figure 5B).

Next, to evaluate the effect of TLR9 on Pg OMVs‐induced IL‐6 mRNA expression, SVG p12 cells were pretreated with 10 µM TLR9 inhibitor E6446 for 30 min, and then treated with Pg OMVs for 2 h. E6446 pretreatment significantly attenuated the IL‐6 mRNA expression increased by Pg OMVs (Figure 5C). These results suggest that the TLR9 pathway, but not the STING pathway, regulates IL‐6 mRNA expression induced by Pg OMVs in SVG p12 cells.

**FIGURE 6:**
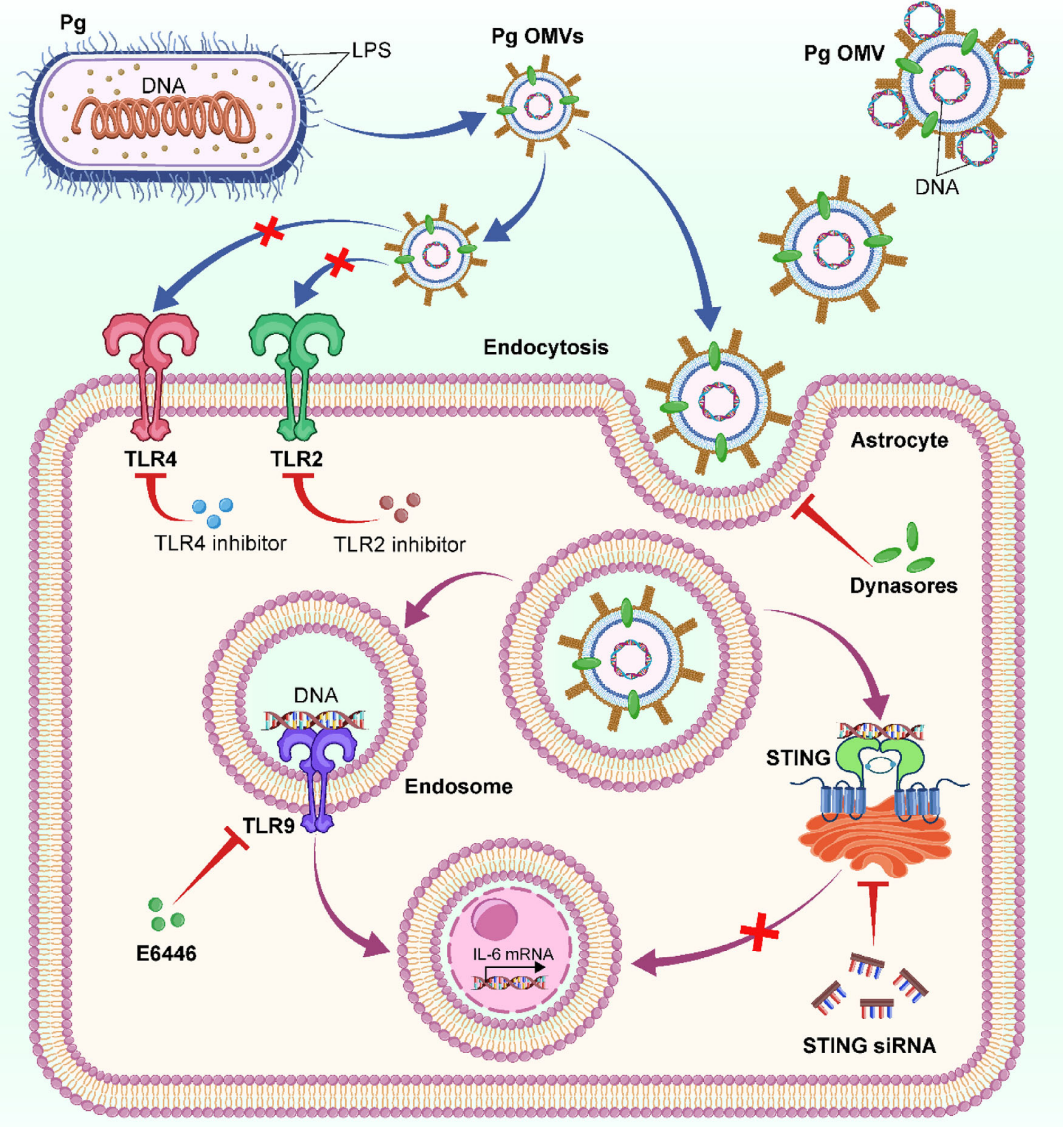
Our proposal model. The periodontal bacteria Pg release OMVs containing Pg genomic DNA. The Pg OMV‐associated DNA is incorporated into astrocytes via endocytosis, but not TLR2 and 4 on cell surface. The incorporated Pg OMV‐associated DNA raises IL‐6 mRNA expression via TLR9 but not STING pathway. This action of Pg OMVs may lead to neuroinflammation and potentially contribute to AD pathogenesis.

In summary, Pg releases OMVs which contain Pg genomic DNA. The Pg OMV‐associated DNA is incorporated into astrocytes via endocytosis and increases IL‐6 mRNA expression via TLR9 (Figure 6). These effects of Pg OMVs may induce neuroinflammation, which could potentially contribute to the onset and progression of AD in periodontal diseases.

## 4 Discussion

This study showed that Pg OMVs can be internalized by astrocytes via endocytosis, which in turn leads to DNA‐dependent inflammatory responses. To our knowledge, this is the first study to demonstrate a relationship between Pg OMVs and neuroinflammation by focusing on DNA. Pg OMVs are transported to various host cell types via endocytosis, and induce pathogenicity. For example, Pg OMVs enter epithelial cells via endocytosis, survive within organelles for extended periods, and impair cellular migration (Furuta, Takeuchi, et al. 2009; Furuta, Tsuda, et al. 2009). Pg OMVs are also endocytosed by endothelial cells, leading to the inhibition of cell proliferation, migration, and angiogenic activity (Chen et al. 2024). Our results showing that endocytosed Pg OMVs induce neuroinflammation are consistent with a previous report demonstrating that endocytosis of Pg OMVs induces the production of inducible nitric oxide synthase (iNOS) and tumor necrosis factor (TNF)‐α by activating pAKT and pJNK pathways in microglia (Chuang et al. 2024).

Pg DNA contains unmethylated CpG motifs which upregulate IL‐6 expression in human gingival fibroblasts (Takeshita et al. 1999) and murine macrophages (Nonnenmacher et al. 2003). These Pg DNA are known to be packaged into OMVs and to bind to OMV surfaces, thereby mediating horizontal gene transfer in Pg strains and contributing to their survival (Ho et al. 2015). However, the role of Pg OMV DNA in host cells remains unclear. In this study, we demonstrated that Pg OMVs DNA were transported into astrocytes by labeling them with a membrane‐permeable DNA stain SYTO 61. The detected DNA was considered Pg genomic DNA, which was sorted into OMVs because the WGS results indicated no differences in DNA between Pg bacteria and Pg OMVs (Figure 2C). These observations suggest that Pg OMV DNA has biological activities similar to those of Pg DNA and that OMVs may play a critical role in delivering bacterial DNA to host cells, triggering pathological conditions.

Bitto et al. (2017) reported that most *Pseudomonas aeruginosa* DNA was present on the external surfaces of OMVs, with smaller amounts located internally. Interestingly, DNA within the internal compartments of *P. aeruginosa* OMVs was consistently enriched in specific regions of the bacterial chromosome encoding proteins involved in virulence, stress response, antibiotic resistance, and metabolism (Bitto et al. 2017). In this study, DNase treatment significantly reduced DNA levels in Pg OMVs, but did not eliminate them (Figure 2D). This suggests that most Pg OMV DNA was present on the OMV surface, with only a small amount found inside. However, it was unclear whether the specific regions observed in *P. aeruginosa* OMV DNA were also present in Pg OMV DNA.

Treatment with DNase significantly suppressed the increase in IL‐6 mRNA levels in Pg OMV‐treated SVG p12 cells (Figure 2E). This finding indicates that the DNA present on the surface of Pg OMVs is involved in IL‐6 mRNA induction. However, IL‐6 mRNA expression was not completely abolished by DNase‐treated Pg OMVs, indicating that DNA within Pg OMVs that is not degraded by DNase may also play a role in the induction of IL‐6. We concluded that DNase effectively removed DNA from the Pg OMV surface based solely on DNA measurement before and after treatment. To further validate this conclusion, immunogold electron microscopy is necessary to observe DNA distribution of DNA on the Pg OMV surface (Tsering et al. 2024). Furthermore, to elucidate the role of Pg OMVs in inducing IL‐6 mRNA expression as demonstrated in this study, it is necessary to distinguish between surface‐bound and internal DNA and to investigate their respective functions.

This study focused on TLR9, an intracellular pattern‐recognition protein that recognizes CpG‐rich DNA (Hemmi et al. 2000), and the STING pathway, a sensor that detects double‐stranded DNA (Xia et al. 2016), as receptors and sensors that can detect intracellular bacterial DNA and induce inflammation. Examination of the TLR9 inhibitor and STING siRNA showed that Pg OMVs DNA enhanced IL‐6 mRNA expression via TLR9 but not via the STING pathway (Figure 5C,D). It has been reported that DNA from the periodontal pathogens Pg, *Actinobacillus actinomycetemcomitans* and *Peptostreptococcus micros* enhances IL‐6 and TNF‐α production in macrophages and dendritic cells via TLR9 (Nonnenmacher et al. 2003). Sahingur et al. (2010) also indicated that DNA from Pg and *Tannerella forsythia* significantly increased cytokine production such as IL‐1β, IL‐6, and TNF‐α by activating the NF‐κB signaling pathway downstream of TLR9 in human monocytic cells (Sahingur et al. 2010). Therefore, TLR9 knockdown experiments should be conducted to verify whether Pg OMV DNA enhances IL‐6 mRNA expression through a similar pathway.

In contrast, a previous study showed that Pg OMVs enhanced IL‐6 expression via the STING and mitogen‐activated protein kinase (MAPK) pathways in human gingival epithelial cells (Uemura et al. 2022). The reason for this discrepancy between these findings and those of the present study remains unknown. It is necessary to verify whether this is due to cell specificity or the conditions under which the Pg OMVs are treated. Furthermore, it remains unclear how DNA from Pg OMVs, once incorporated into SVG p12 cells via endocytosis, binds to and acts on TLR9 on the endosomal membrane. These points should be clarified in future studies.

Our research model had several limitations. Only one cell line (SVG p12) was used to confirm the induction of IL‐6 expression by Pg OMVs. To validate the conclusions of the present study, it is necessary to confirm whether similar results can be obtained in other cell types, such as astrocytes isolated from the brain tissue. Another limitation is that only the TLR and STING pathways were analyzed as mechanisms of IL‐6 induction. The expression of IL‐6 in astrocytes is regulated by various factors including cytokines, infectious agents, neuropeptides, and neurotransmitters, most of which act synergistically (Van Wagoner and Benveniste 1999). For instance, TNF‐α and IL‐1β have been reported to induce IL‐6 expression in rat astrocytes. Furthermore, inducible IL‐6 can enhance its own expression through autocrine positive feedback by activating the Janus kinase (JAK)/signal transducer and activator of transcription 3 (STAT3) and MAPK pathways (Van Wagoner et al. 1999). Further investigation is needed to determine whether Pg OMVs induces these factors and whether this could regulate IL‐6 expression.

## Author Contributions

Conceptualization: Kaya Yoshida and Kazumi Ozaki. Data curation: Kaya Yoshida and Yuka Hiroshima. Formal analysis: Yuka Hiroshima. Funding acquisition: Kaya Yoshida. Investigation: Ayu Takai, Kaya Yoshida, Ayu Ikuta, Mariko Seyama, and Yuta Uemura. Methodology: Kaya Yoshida, Yuka Hiroshima, and Yuta Uemura. Project administration: Kaya Yoshida and Kazumi Ozaki. Resources: Yuta Uemura and Hiromichi Yumoto. Supervision: Kaya Yoshida and Kazumi Ozaki. Writing – original draft: Ayu Takai, Kaya Yoshida, and Yuka Hiroshima.

## Funding

This study was supported by a grant from JSPS KAKENHI to Kaya Yoshida (grant number: JP23K09505).

## Conflicts of Interest

The authors declare no conflicts of interest.

## Supporting information




**Supporting File**: omi70030‐sup‐0001‐FigureS1.tif

## Data Availability

The data sets used and/or analyzed during the current study are available from the corresponding author on reasonable request.
