# Supplementary figures and images for: Porphyromonas gingivalis Outer Membrane Vesicles‐Associated DNA Triggers Inflammation by Inducing IL‐6 in Astrocytes
Source: Mol Oral Microbiol. 2026 Apr 1;41(4):227–38. doi: 10.1111/omi.70030 (PMC13325520; doi:10.1111/omi.70030)

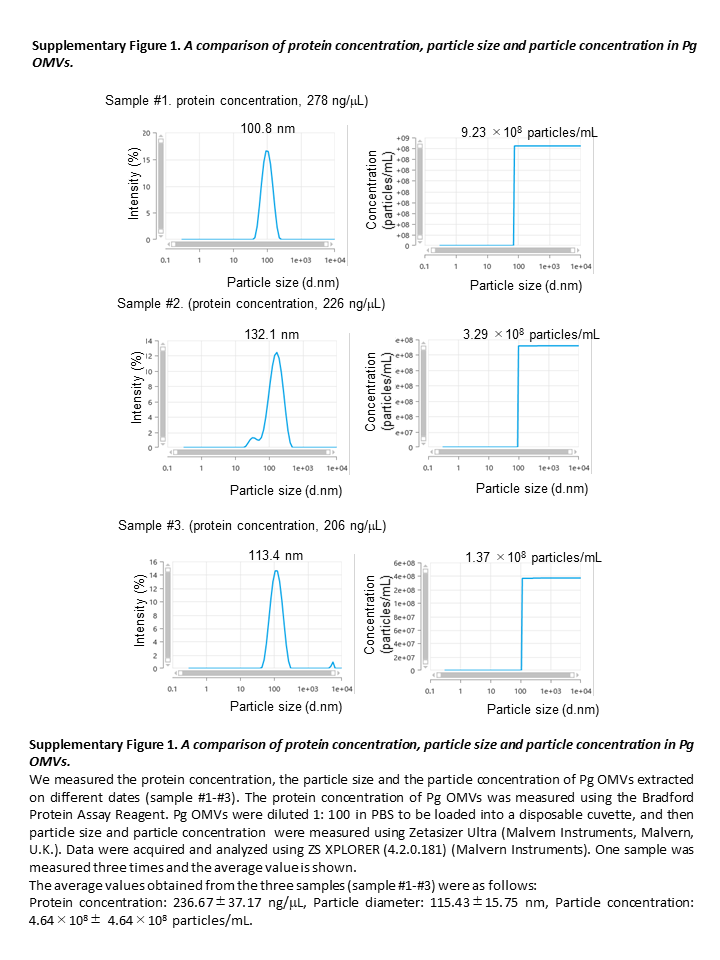

Supplement: Supplementary file 1 — Supporting File: omi70030‐sup‐0001‐FigureS1.tif [file OMI-41-227-s001.tif]
